# Global structures and local network mechanisms of knowledge-flow networks

**DOI:** 10.1371/journal.pone.0246660

**Published:** 2021-02-16

**Authors:** Marjan Cugmas, Anuška Ferligoj, Miha Škerlavaj, Aleš Žiberna

**Affiliations:** 1 Faculty of Social Sciences, University of Ljubljana, Ljubljana, Slovenia; 2 National Research University Higher School of Economics, Moscow, Russia; 3 School of Economics and Business, University of Ljubljana, Ljubljana, Slovenia; 4 Department of Leadership and Organizational Behavior, BI Norwegian Business School, Oslo, Norway; University of Lausanne, SWITZERLAND

## Abstract

Understanding the patterns and underlying mechanisms that come into play when employees exchange their knowledge is crucial for their work performance and professional development. Although much is known about the relationship between certain global network properties of knowledge-flow networks and work performance, less is known about the emergence of specific global network structures of knowledge flow. The paper therefore aims to identify a global network structure in blockmodel terms within an empirical knowledge-flow network and discuss whether the selected local network mechanisms are able to drive the network towards the chosen global network structure. Existing studies of knowledge-flow networks are relied on to determine the local network mechanisms. Agent-based modelling shows the selected local network mechanisms are able to drive the network towards the assumed hierarchical global structure.

## Introduction

It is well known that the various global network structures found within a company not only influence how knowledge is created by giving individuals opportunities to access and combine knowledge [1], but also affect an individual’s willingness and ability to transfer more complex knowledge [2]. While many studies have considered different global network structures, less is known about which local network mechanisms promote their emergence.

Among the many definitions of local mechanisms [3], Stadtfeld [4] relied on, Coleman’s [5] macro-micro-macro model places local network mechanisms in three classes: situational mechanisms, action-formation mechanisms, and transformational mechanisms. These three local network mechanism types can be used to explain a central puzzle in both sociology and social network research: How do micro-level network processes generate macro-level network structures? [6].

The paper addresses the question of whether the selected action-formation type mechanisms and transformational types of local network mechanisms can lead a network towards a network with the global network structure proposed in this paper. The global network structure is selected following a discussion on the characteristics of global network structures of knowledge-flow networks and analyses of empirical knowledge-flow networks (section Global network structure and knowledge flow). The choice of local network mechanisms is based on the theory of Nebus [7] (section Local network mechanisms and knowledge flow).

A possible model to explain the proposed micro-macro link is proposed in this paper, although it is possible that other models might also explain it (including, e.g., different local network mechanisms and nodes’ attributes). However, knowing whether the selected network mechanisms can lead to the proposed global network structure is important for several reasons. One reason is for planning research on empirical knowledge-flow networks, while another is to understand the link connecting local network mechanisms and global network structures which is vital for companies or other organizations seeking to develop policies to promote the emergence of a targeted global network structure.

The research question of whether the selected local network mechanisms can produce the chosen global network structure is addressed in section Generating networks with a hierarchical-cohesive blockmodel by using an algorithm from the family of Network Evolution Models (NEM) [8], which allows networks to be generated by considering the selected local network mechanisms.

The results are discussed and summarized in the last section along with the main assumptions used in this study.

## Global network structure and knowledge flow

This section aims to discuss which global network structures might be the most appropriate for knowledge transfer according to different studies on knowledge flow. This global network structure is then tested on an empirical example.

In this study, the units in the network represent employees while the links between them operationalize the transfer of knowledge. The global network structure can be described with a blockmodel to help reduce the empirical network’s complexity [9] and as a tool to operationalize social roles [10, 11].

A blockmodel is a network in which the units (nodes) are clusters of equivalent units within a studied network. The term “block” refers to a submatrix of the network that reveals the relationship between two clusters or within a single cluster [9]. In the case of structural equivalence, only two block types are possible, namely, the complete block type (with links between each unit from the first cluster and each unit from the second cluster) and the null block type (without any links between first-cluster units and second-cluster units) [12]. The second cluster can be the same as the first one (a diagonal block).

**Fig 1:**
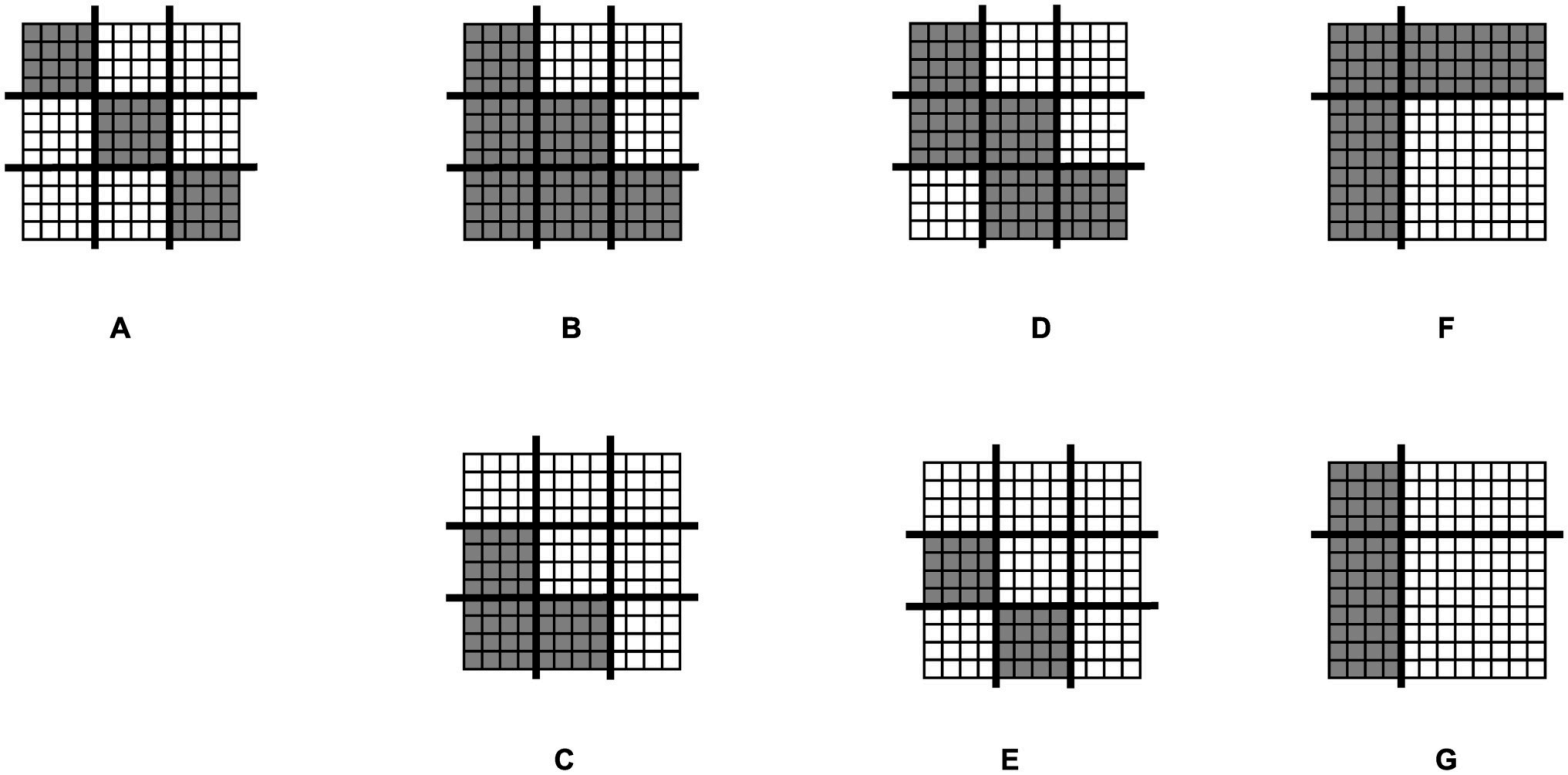
Networks with the most common blockmodel types. The units are ordered by rows and by columns in line with a given blockmodel, the clusters are separated by thicker lines. A: Cohesive. B: Transitive-cohesive. C: Transitivity. D: Hierarchical-cohesive. E: Hierarchical. F: Symmetric core-periphery. G: Asymmetric core-periphery.

The most commonly studied blockmodel types are: cohesive, symmetric or asymmetric core-periphery, hierarchical, and transitivity. The last two can be defined with (i.e. a transitive-cohesive blockmodel and a hierarchical-cohesive blockmodel) or without (i.e. a transitivity blockmodel and a hierarchical blockmodel) links between units in the same cluster. A matrix representation of networks drawn in line with these blockmodels is shown in Fig 1.

Authors often use terms like “cohesive”, “central”, “core-periphery” or “hierarchical” to describe a global network structure. These terms are frequently defined and operationalized in very dissimilar ways. Therefore, two very similar global network structures might be described in two very distinct ways, and vice versa.

The terms “central” and “hierarchical” describe two global network properties that are usually dependent but may relate to very different global network structures from the blockmodeling point of view. For example, the core units in a core-periphery blockmodel type are central and hold the highest position in the network hierarchy. In contrast, the hierarchical or transitivity blockmodel entails a high level of hierarchy where the units in the top cluster are also the most central. These blockmodels are quite distinct and probably have a different impact on how knowledge is transferred within a company.

### Blockmodel type and knowledge flow

This subsection broadly overviews studies that discuss different global network properties in relation to work efficiency or knowledge transfer efficiency. Although various sociological concepts are used to explain this relationship, they will be used in the discussion on recommendations for different global network structures for certain types of knowledge transfer.

It is shown that a highly centralized global network structure (e.g., core-periphery blockmodel type or transitivity blockmodel type) can negatively affect the transfer and performance of knowledge. This is because being central in an advice-giving network may provide a central individual with greater prestige, in turn leading to an overload of requests for advice from others. In order to avoid this overload, the most central units start to refer advice-seekers to other units in the network. This requires fresh coordination between the most popular units so as to avoid status competition or conflicts [13]. Further, maintaining a high number of social ties can lower well-being [14] and might become so demanding that it reduces work performance [15, 16].

A global network structure consisting of several non-linked cohesive groups (e.g., cohesive blockmodel type) might not be optimal for group performance. For example, Sparrowe et al. [17] showed that leaders of groups that are more centralized (measured by degree centrality) estimate the performance of their group as lower. Wong [18] contended this might relate to the variety of knowledge, which is smaller in more centralized groups. To avoid this, a manager might wish to develop policies to promote the sharing of knowledge outside groups. This is particularly important when groups are structurally more diverse or possess different kinds of knowledge since members can benefit from different knowledge sources external to their group [19], although members of different groups might find it difficult to transfer knowledge (especially with respect to more complex tasks or knowledge) because communicating between them could prove too strenuous [20].

The nodes or groups of nodes which link different groups are called bridging units or groups [21]. If bridging units or groups are linking several cohesive groups in such a way that the average path length is short, this structure is usually called a small world structure [22, 23]. It is believed that bridging units or groups of units enable knowledge to be transferred among different groups of units. Studies show that this depends greatly on the complexity of the knowledge. When knowledge is simple, the presence of a bridge is a necessary and sufficient condition for knowledge transfer, yet more complex knowledge is likelier to be transferred (across bridging units or groups) in the presence of bridging when individuals have either a strong tie to both groups or a diverse network [2]. Namely, more complex knowledge is more likely to stay embedded within local communities of practice [2]. In terms of group productivity—the most productive teams are internally well connected but have many links to the other groups [24]. It is also hypothesized that bridging units or groups have a greater capacity to observe new knowledge than the others [2].

No single global network structure exists to apply to knowledge transfers within any type of organization because a large number of factors are at play [25]. However, in the review of literature on this topic in the previous section one finds some very general guidelines on global network structures.

When tasks are very complex, promoting the establishing of links between units from the same task group (or business unit) is recommended. Here, establishing non-formal relationships is particularly important. In order to further expand the variety of knowledge available to the group, the manager should consider promoting bridging units/groups between groups. To avoid overload of an individual or a group, the number of connections should be limited and hence also the number of bridging units/groups. It is suggested that a unit or a group should bridge only those who are not too different with respect to their knowledge.

According to Lazega [26], “smaller knowledge-based organizations should have a structure of relationships closer to cohesive groups, while large (mainly manufacturing) systems are supposed to look similar to hierarchical blockmodels”.

Based on the above, it appears that a hierarchical-cohesive blockmodel should be the most efficient for intra-company knowledge transfer. When the knowledge is more complex, the emphasis should be on the links within groups and the bridging cores since it is observed that groups working on more complex problems naturally tend to develop less central communication patterns and groups faced with less complex tasks tend to develop more centralized communication patterns [27], whereas when the task or knowledge is simpler the stress should also be placed on the hierarchy’s emergence.

**Fig 2:**
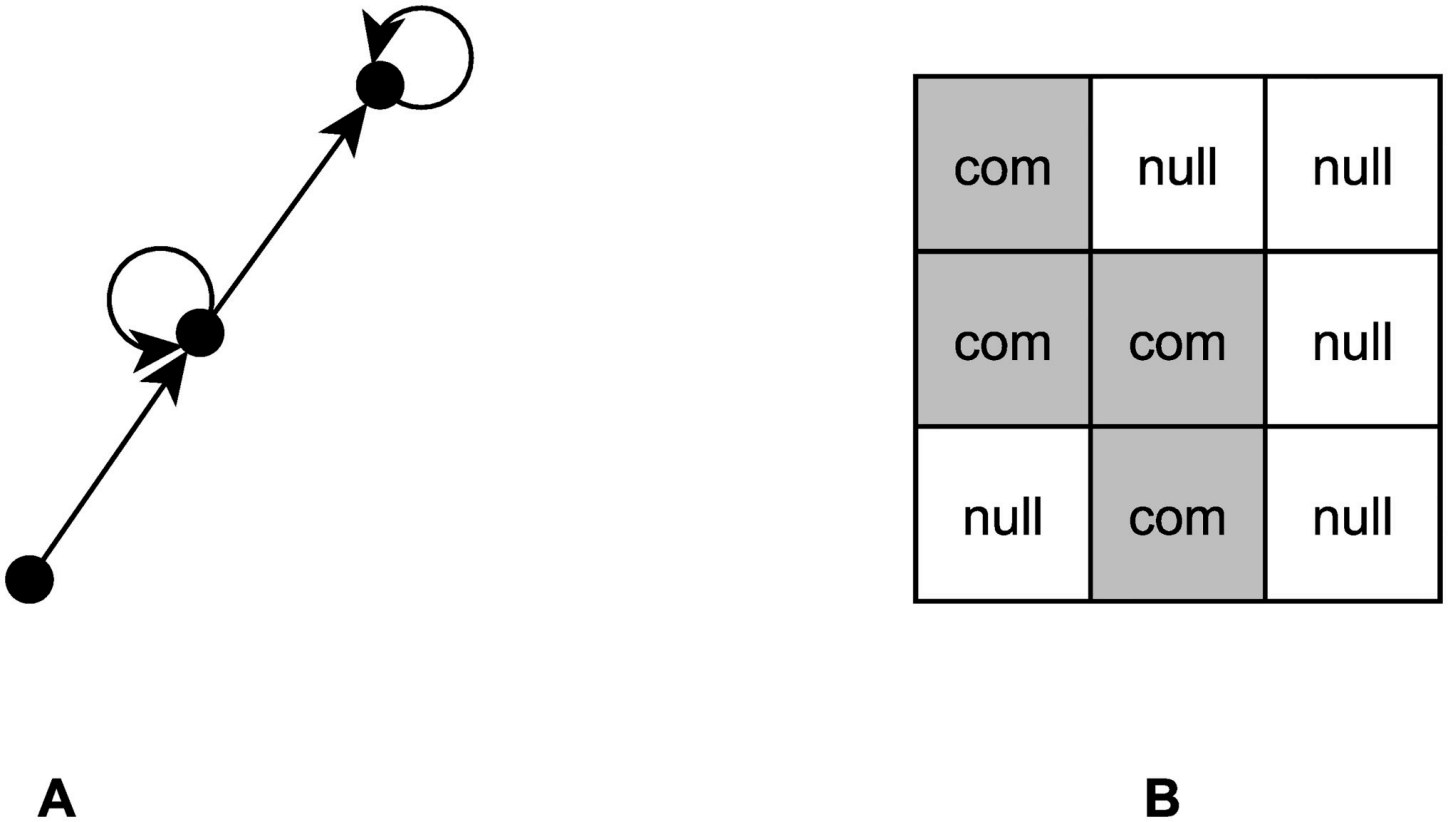
The chosen hierarchical-cohesive blockmodel with the last non-cohesive group. A: graph representation. B: matrix representation.

In a growing company, it can happen that the latest newcomers are not yet connected to each other. Therefore, in the hierarchical-cohesive blockmodel, the units of the last cluster are not connected to each other, i.e. it is assumed that the last diagonal block is a null block. The selected blockmodel type of interest in this study is therefore a hierarchical-cohesive blockmodel with the last diagonal block of the null type (shown in Fig 2), meaning there are no links among the units on the lowest hierarchical level. Three clusters are assumed.

### Blockmodel type in a knowledge-based company

Since the hierarchical-cohesive blockmodel with the last non-cohesive group was proposed as potentially efficient for intra-company knowledge flow, the secondary network data (data in S1 Data) are analysed in this subsection to verify whether this blockmodel type appears in a real social context.

The data were collected in a Slovenian knowledge-based company during the company’s growing stage. The latter is important to note since the global network structure is evolving very rapidly in this stage and thus allows a better study of the underlying local network mechanisms (which are different than in a well-established large company). The chosen company is a typical representative of such company types, except for a higher number of business units, which is to be taken into account while identifying the global network structure of the knowledge-flow networks.

These data were already analysed using different approaches, such as exploratory data analysis and Stochastic Actor-Oriented Models (SAOM). Especially the latter was used to test which local network mechanisms affect the establishing and dissolving of ties in this empirical knowledge-flow network [28–30], but not to study the global network structure by the appropriate blockmodel type, which is the aim of this subsection.

#### Company profile and data collection technique

The data were collected at three points in time (December 2004, July 2006, April 2007) in a company whose core business is software development, IT and business consulting, maintenance and support (Skerlavaj 2007).

The company was founded in 1989 with a subsidiary established in Croatia in 2000 and a joint venture in Serbia in 2003. In this research, the focus is on the employees who work in Slovenia (Ljubljana) since geographical location has a very prominent impact on the global network structure (i.e. there are almost no links between the employees from different countries) [28–30]. The number of survey participants was increasing over the years of collecting the data (December 2004: 59; July 2006: 60; April 2007: 80), reflecting the fact the company was growing (Table 1).

**Table 1:** Size of the business units.

Time of collecting the data		Business Units					Total
		Common services	Navision	Industry solutions	Banking solutions	Directorate	
December 2004	Frequency	4	22	16	11	2	55
	Percentage	7	40	29	20	4	100
July 2006	Frequency	4	29	13	12	2	60
	Percentage	7	48	22	20	3	100
April 2007	Frequency	10	30	22	15	3	80
	Percentage	13	38	28	19	4	100

The totals might differ from the total number of participants due to some further missing values.

Three business units were operating: Enterprise Resource Planning Solutions (Navision), Industry Solutions, and Banking Solutions. The employees of the common services and the directorate were also included in the analysis. Most employees worked in Navision (at all three time points). This is also the business unit in which most new employees were employed.

At all three points in time, approximately 75% of the workers were male. The average tenure (number of months employed by the company) was 48 (*sd* = 42) in December 2004, 53 (*sd* = 42) in July 2006 and 41 (*sd* = 34) in April 2007. Half the employees had worked for 42 months with the company in December 2004 and in July 2006, while in April 2007 half the employees had been working for the company for only 26 months. This is due to many new employees arriving between the last two observations.

**Fig 3:**
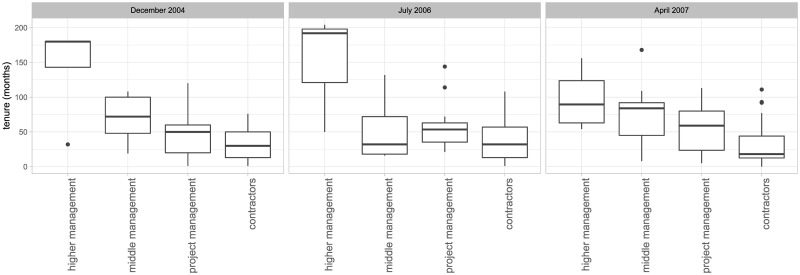
Relationship between tenure and hierarchical position in the company.

Most employees were contractors (56%—64%) or project management (20%—28%) while the minority was in higher or middle management. In April 2004, a very clear relationship existed between tenure and hierarchical position in the company, while at the latter time points this relationship became less clear (Fig 3).

Different name generators were used to measure the knowledge flow in the company. In this study, only those that were used at all time points are considered: (i) “Who in the company do you ask when you need advice or information related to work?”; and, (ii) “Who are the others in the company from whom you learn the most?”. The employees were allowed to list as many others as they wanted. Instead of names, they used codes that had been already assigned to each employee in order to ensure confidentiality. One or two employees listed all of their co-workers from the same business units. Such answers were considered as valid.

**Fig 4:**
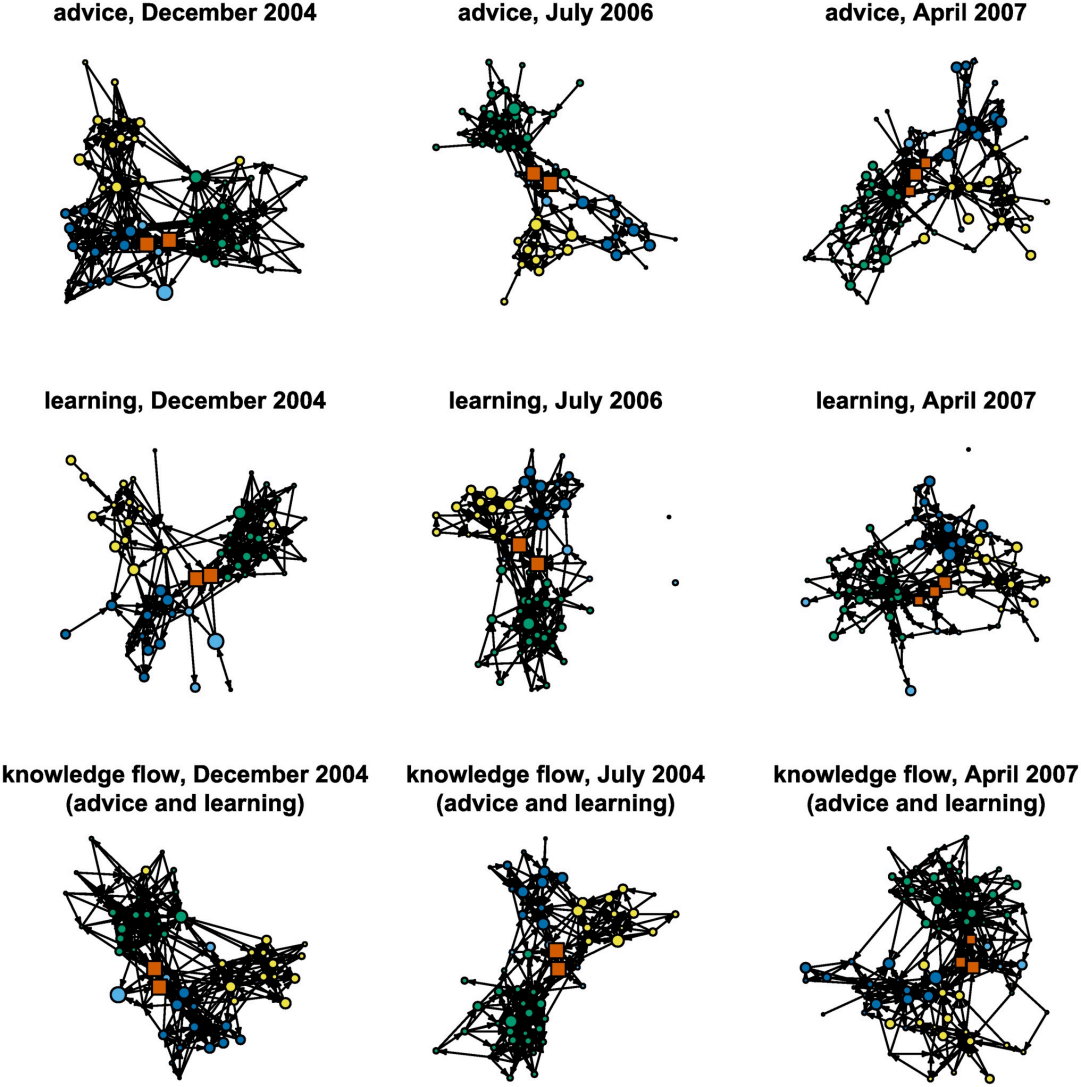
Binary advice, learning and advice and learning networks for different points in time.

The asymmetric binary complete networks were created based on the information obtained (Fig 4). The networks are sparse (with a density of between 0.03 and 0.10 for all networks). The learning networks are generally sparser than the advice networks. This might be due to the perception of advice and learning. Giving advice may be seen as less formal and less threatening in the sense of possibly losing one’s non-formal hierarchical status in the company, compared to learning, which is more status-related. The density of the networks is also decreasing over time in both network types, which may show the networks are growing in time. In order to retain the same density as the network grows, the average in-degree or out-degree must increase. However, in the empirical networks under study the mean in-degrees and out-degrees are slightly lower at later points in time, possibly showing that it takes time for newcomers to develop their personal learning and advice networks in the company.

Clusters are seen to form in all networks in the visualizations shown in Fig 4. The size of the nodes is proportional to employee tenure while different colors denote different business units. The clusters are mainly separated by the business units, which is expected since the different business units deal with very specific areas of work. There are also two or three bridging nodes (rectangular shapes in Fig 4) in almost every network. These nodes have a higher tenure and belong to the directorate.

The global network structures are similar in both the advice network and the learning network because the two name generators are measuring the same dimension, i.e., knowledge-flow. Therefore, the advice and learning networks are combined to form so-called knowledge-flow networks. A link from unit *i* to unit *j* exists in the knowledge-flow network if it exists in the advice network or the learning network. The density of the knowledge flow decreases from 0.11 in December 2004 to 0.05 in April 2004.

#### Methodology

Generalized blockmodeling for sparse networks [31] is used to obtain a blockmodel. Blockmodeling is a procedure for reducing large, potentially incoherent networks to smaller, more comprehensible and interpretable structures [9]. With generalized blockmodeling, the solution is obtained by a relocating algorithm that minimizes the value of a criterion function [12, 32, 33]. A criterion function reflects inconsistencies (the differences between the empirical and ideal solution).

Using the generalized blockmodeling on the empirical networks revealed the approach is problematic when relatively sparse binary networks are being analyzed. When structural equivalence is used, only very small complete blocks are typically found [31]. Therefore, several approaches to blockmodeling sparse networks were developed. One approach is by differently weighting the inconsistencies of null and complete blocks. Žiberna [31] suggested the weight *d*/(1 − *d*) for complete blocks and 1 − *d* for null blocks, where *d* is the density of the whole network. This approach finds denser and sparser blocks without being limited to perfectly null and perfectly complete blocks.

The number of random restarts in the relocating algorithm is set to 500 and the number of clusters is estimated based on the dendrograms (Ward’s agglomerative clustering method is used on a dissimilarity matrix obtained by corrected Euclidian distance [9]) and the stability of the blockmodeling solution in time, which is assessed by a visual examination of the networks and blockmodels.

#### Results

**Fig 5:**
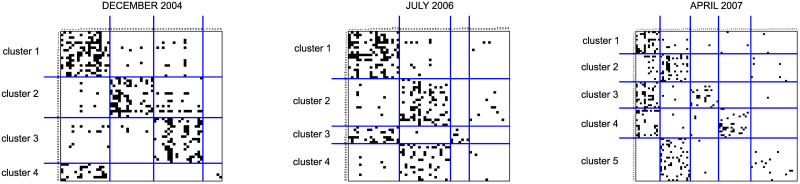
The knowledge-flow networks drawn in matrix form in line with the blockmodeling solution.

The blockmodeling solutions for the knowledge-flow networks at the three time points are shown in matrix form in Fig 5. The employees are listed by rows and by columns and the order of the rows and the columns is consistent with the blockmodeling solution. Each dot represents a link. Blue lines distinguish the clusters that are obtained.

There is a relatively small number of inconsistencies in complete and in null blocks in all three solutions. The global network structure of the first network is close to the cohesive one with three clusters. However, there is a cluster of seven employees who are well linked to the first cohesive cluster, but are internally not linked to each other. This part of the network expresses the tendency for an asymmetric core-periphery structure in the network. The global network structure of the second network consists of two separate parts where each part’s structure is asymmetric core-periphery (1^st^ and 3^rd^ cluster, 2^nd^ and 4^th^ cluster). The diagonal block, corresponding to the third cluster, is classified as a complete block even though only a few links are present.

The tendency towards a hierarchical-cohesive blockmodel is expressed in the network, observed at the last time point. The units from the fifth cluster are linked to the units in the second cluster while the units in the second cluster are weakly linked to the units in the first cluster. The units from all clusters, except the fifth, are internally well linked. The third and fourth clusters are linked to the first cluster.

The similarity or stability of the obtained clusters may be evaluated using the Rand Index [34] or Modified Rand Index [35]. Both indices are defined based on the number of pairs of units that are classified in the same or different clusters in both partitions. The Rand Index requires that two partitions to be compared are obtained on the same set of units. If newcomers are outgoers are present, they must be removed from the data prior to analysis. On the other hand, the Modified Rand Index is defined such that newcomers and outgoers lower the index value, along with the merging and splitting of clusters. Non-adjusted measures can take values on the interval between zero and one, where a higher value indicates more stable or similar partitions. In general, they are not comparable and are therefore adjusted for chance. In that case, the indices’ expected value equals around zero in the case of two random and independent partitions and for the case of two identical partitions the values of both indices equal one.

The values of the indices are given in Table 2. The Rand Index indicates that the clusters which are obtained are relatively stable when only those present in both time periods are considered. On the other hand, the stability of the clusters is extremely low when newcomers and outgoers are also considered, as is the case in the company under study. A high level of dynamics is expected since the global network structure was observed in the growing stage of the company.

**Table 2:** Stability/Similarity of the obtained clusters—Adjusted indices.

	December 2004 vs. July 2006	July 2006 vs. April 2007	December 2004 vs. April 2007
Rand Index	0.43	0.32	0.21
Modified Rand Index	-0.01	-0.08	-0.08

**Fig 6:**
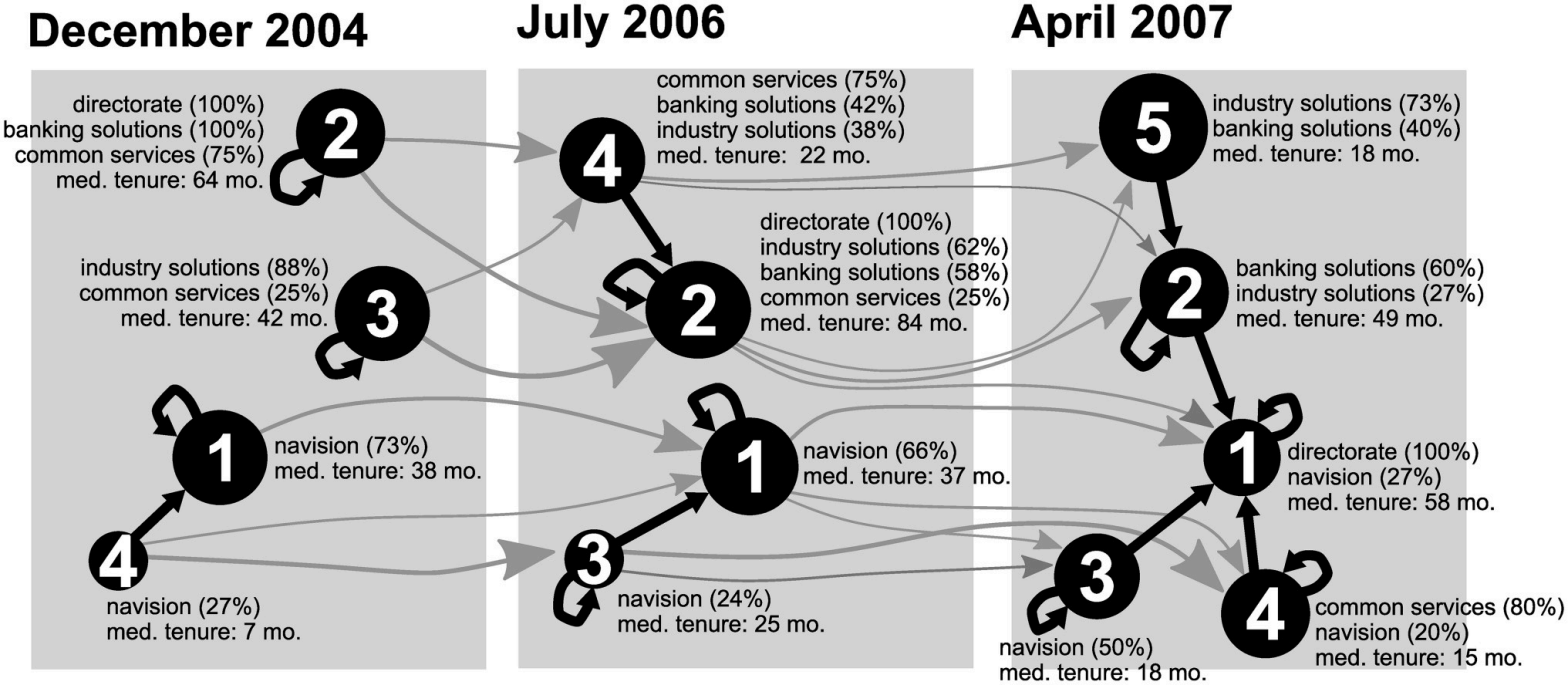
Stability of the clusters obtained by blockmodeling and their structure (for the business units).

Fig 6 visualizes the structure and stability of the clusters in time. The nodes represent clusters. Loops show that the employees within the clusters are well linked. The sizes of the nodes are proportional to the number of employees classified in each node. Black arrows visualize the relationships between the clusters. Gray arrows show the selected transitions between clusters over time. The numbers of nodes for the IDs of the clusters correspond to Fig 5. The percentage of employees from each business unit and the median number of months having worked for the company (tenure) are also given. These are important differentiators between the various clusters. Based on the Fig 6, it seems the initial network structure was mostly determined by expertise (i.e. by business units). However, those with a lower tenure tend to acquire knowledge from the more experienced employees. As time passes, the directorate (along with some “old-timers”) becomes more and more central. The very peripheral clusters (which are internally non-linked at the start) mostly consist of newcomers. Many newcomers became outgoers at the later time. Yet, newcomers tend to ask for advice from those who are more experienced and come from a similar business unit.

The global network structure of the empirical knowledge-flow network is changing dramatically from (similar to) the cohesive and core-periphery blockmodel to the blockmodel very close to the hierarchical-cohesive one with the last non-cohesive group.

## Local network mechanisms and knowledge flow

It was shown that the blockmodel type, close to the hierarchical-cohesive blockmodel type with last non-cohesive group, appears in a real company. While SAOM and ERGM have been used to study knowledge-flow networks, they can used to model the establishing and dissolving of ties on a micro level. However, they cannot be explicitly used to model a global network structure (e.g. in terms of a blockmodel).

It is known that the micro mechanisms can include different types of network-related mechanisms and different units’ attributes. In this paper, we plan to study if only network-related mechanisms can produce the proposed blockmodel type. Therefore, the set of possible local network mechanisms is narrowed to the mechanisms that do not include the units’ attributes (i.e. the ego does not have any information about the units’ attributes). The exception is so-called “tenure”, which is an endogenous attribute (i.e. it can be estimated from the network). The selection of the local network mechanisms is discussed in this section.

The local network mechanisms are selected according to the theory [7] of network formation for predicting a given unit’s selection and retention of forming an advice network. Although the theory assumes ego-centred networks, the well-developed propositions are used in the context of full networks in this study.

The theory assumes that the ego who is seeking help may possess very detailed information about potential contacts (the contact-information-rich case) or no information on potential contacts (the contact-information-poor case). In the former scenario, the ego can compare the net knowledge value of all potential contacts before choosing one while, in the latter case, the ego does not possess any decision-relevant information. However, as the knowledge held by the ego is increasing, the potential of having information about contacts (experts) is also increasing.

Several mechanisms are considered in this study. They are summarized in Table 3 and described in the following subsections.

**Table 3:** The considered local network mechanisms.

		NAME OF THE OPERATIONALIZATION OF THE MECHANISMS	MECHANISMS	OPERATIONALIZATION OF THE MECHANISMS
**PV**		**ALTER-BASED MECHANISMS**		
		Hierarchical position of the alter	expertise	how many nodes can reach a given unit
		Tenure of the alter	experiences, skills	tenure (time in the network)
		Popularity level of the alter	willingness to share knowledge, cognitive trust	in-degree
		**DYAD-BASE MECHANISMS**		
	**PC**	Outgoing shared partners	cognitive distance, realizing the alter’s knowledge value	number of outgoing shared partners between the ego and the alter
		Difference in hierarchical position between the ego and the alter	social cost, psychological cost, institutional cost, organizational separation	the difference between the number of nodes that can reach the ago and the number of nodes that can reach an alter
		Difference in tenure between the ego and the alter	likelihood of a response, trust	difference in tenure between the ego and the alter
		Distance between the ego and the alter	psychic distance, cognitive distance, geographical distance	geodesic distance between the ego and the alter

PV: Perceived value. PC: Perceived cost.

### Mechanisms related to the perceived value of alters’ advice

The four mechanisms (i) hierarchy, (ii) tenure, (iv) popularity and (iii) number of other units to which both the ego and the alter have a tie (Outgoing-Shared-Partners, OSP) may be related to the perceived value of the alters (all, except OSP mechanisms, are also independent of the ego). OSP is also considered to be related to the perceived cost of asking for advice.

It is assumed that those in a higher hierarchical position possess greater expertise while those with a higher tenure might possess more experience and skills for independent problem-solving. Those with a higher tenure are more often formal or non-formal mentors to the newcomers. Hierarchical position and tenure can be dependent.

The most popular ones are those with the highest in-degree. They are seen (by the ego) as the most active and are therefore perceived (by the ego) as being the most willing to share their knowledge. The cost of obtaining knowledge from such units is accordingly perceived to be lower. However, being in receipt of many requests can become a burden for the most popular ones such that the probability the ego will be given actual high-quality help might be lower than perceived.

### Mechanisms related to the perceived cost of asking for advice

The mechanisms in this subsection relate to the ego’s perception of the cost of the advice given by potential alters. The perceived cost not only depend on the alter, like occurs more with the value-related mechanisms, but also on the ego. Therefore, one can say that four mechanisms describe the ego-potential alter relationship: (i) difference in hierarchical position in the network; (ii) difference in tenure between the ego and the alter; (iii) distance between the ego and the alter; and (iv) OSP.

These mechanisms are generally related to the perceived cost of asking for advice and the probability that the alter will adequately respond to the ego. For example, the probability the selected alter will accept the request for advice depends on the difference in their hierarchical levels, and is decreasing when the absolute difference is increasing. Responding to those on a much lower hierarchical level might bring a risk with high social cost (e.g., loss of social status) for those in the higher hierarchical position. Also asking for advice from those on a much higher hierarchical position can entail a risk with high psychological cost (e.g., inability to formulate the problem, stress from fear of rejection) for those on the lower hierarchical level. For both, the institutional cost (e.g., formal or non-formal feedback can follow after passing formal processes or lines of authority) can be high.

It is reasonable to assume that the distance (e.g. shortest path) between the ego and the alter is negatively associated with the probability the alter will respond to the request for advice. The distance between the ego and the alter can be associated with the geographical, psychic (reflecting cultural and institutional differences) or cognitive distance between them, which increases the cost and opportunities of contacts.

The number of partners shared by the ego and alter as defined above operationalizes the cognitive distance and the ego’s ability to realize the value of the alter’s knowledge. Therefore, it is considered as a cost-related mechanism as well as a value-related type of mechanism.

## Generating networks with a hierarchical-cohesive blockmodel

The main research question is addressed in this section, i.e. whether the selected local network mechanisms can drive the network towards a hierarchical-cohesive blockmodel type with the last non-cohesive group. This means that the aim is not to test which local network mechanisms generated the empirically observed network discussed before.

To address the research question, many networks are generated using the proposed NEM algorithm described in the following subsection. The algorithm (see the next subsection and S1 Appendix for details) considers the local network mechanisms selected in the previous section and described in greater detail in the Local network mechanisms and their weights subsection. Different weights are applied to the operationalized local network mechanisms.

Depending on the local network mechanisms being considered, it is assumed that all units in the network are familiar with the global structure of the network and some of the units’ attributes (i.e. tenure in this study). This assumption is reasonable in the case of smaller networks with existing (formal or non-formal) communication between the units.

To simplify the model, it is also assumed that the units have an equal probability of establishing a link (all employees are equally likely to e.g. ask for advice). The future development of the approach could also consider non-equal probabilities of establishing a link.

### Algorithm for generating networks

The network is represented in the form of an adjacency matrix *X* with *n* rows and *n* columns, both corresponding to the number of units n in the network. The 1s in this matrix represent links from row units to column units and 0s represent non-links. Each mechanism described in the Local network mechanisms section is operationalized by an appropriate statistic (see the next subsubsection).

Before the iterative algorithm is applied, the user must specify the initial network (besides the mechanisms and their weights). Parameters λ and *κ* must also be set. Parameter λ expresses the maximum expected out-degree, while parameter *κ* is related to the number of iterations. The actual number of iterations *k* is calculated based on the size of the network *n*, parameter λ and parameter *κ* (see S1 Appendix). The local network mechanisms must also be provided with the corresponding vector *θ* which operationalizes the importance (strength) of the selected local network mechanism.

According to the characteristics of the company considered in this study, the initial networks are random networks of size 30 units. In these networks, each link exists with a probability of 0.25. Parameter λ is set to 6 based on the average degree in the empirical networks analyzed in this study while parameter *κ* is set to 4, which is a compromise between the convergence of the global network structure before the new nodes are added to the network and the (computational) time needed to generate the networks. The total number of iterations *k* is 4,320.

At each iteration of the algorithm, a unit (ego *i*) is selected among all the units in the network. Each unit is selected with an equal probability (i.e., it is assumed that all the units have an equal probability to ask for advice or to learn from another at any time) in this study (yet it could be assumed that those units with a lower tenure will have more opportunities to ask for advice; whether this is a reasonable assumption depends on the company’s policies and organizational culture).

Considering unit *i* and the selected local network mechanisms, the mechanisms’ statistics are calculated for each unit and also weighted by *θ*. The weighted statistics are normalized into the interval between 0 and 1. Among 25% of the units with the highest normalized weighted statistics value, a unit *j* (alter) is randomly selected. The tenure, which is an attribute value on each unit, is calculated upon each iteration (tenure is precisely defined in S1 Appendix). Some new units (newcomers) are added to the network and some old units (outgoers) are removed from the network at the selected iterations.

**Fig 7:**
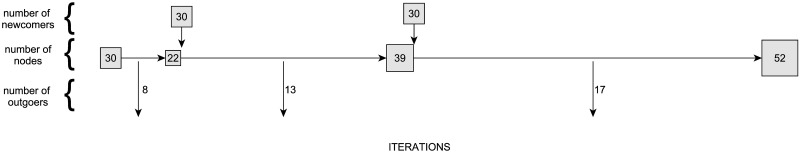
Size of networks at different numbers of iterations, newcomers and outgoers.

The units are added to the network in waves. The time points at which they are added to the network are predefined (see Fig 7). The new units do not have any prior link at the time they are added to the network. In this study, 30 units are added to the network at the 720th iteration and 30 units are added at the 2,160th iteration. The number of iterations between two waves is set in such a way that all the units have at least λ*κ expected opportunities to set a link. The duration of the links is the number of iterations between the two consecutive waves devided by *κ*.

The number of outgoers is selected arbitrarily (25% out of all the units just after a given wave of newcomers is added to the network). With this implementation of the algorithm, the outgoers leave the network (usually one by one) at the randomly selected iterations. The results (not included here) of a preliminary analysis from the empirical data show that the main personal and network characteristics of the units do not differ between the outgoers and the others. Therefore, in this study the units to be removed (outgoers) are selected randomly. Before the 1^st^ wave of new units, 8 already existing units leave the network. Between the 1^st^ and 2^nd^ waves of new units, 13 units leave the network. Between the 2^nd^ wave and the end of the iterations, 17 units leave the network.

### Local network mechanisms and their weights

As proposed by Cugmas et al. [36], “the term mechanism describes a process that drives the concrete actions by units in the network (e.g., creating a link to a highly-popular unit)”. The mechanisms are operationalized by appropriate statistics that reflect the mechanisms. These statistics are used in the proposed NEM as described in the previous subsections and are as follows:

- **Tenure of the alter** and **difference in tenure between the ego and the alter**: The relative value of tenure *t* (which is a vector of length *n* as the nodes’ attribute) is calculated for the *i*-th unit as
  $$\begin{array}{c} RT\left(i\right)=\frac{t_{i}}{\frac{1}{n}\sum_{l=1}^{n}t_{l}} \end{array}$$ (1)
  while the difference in tenure between unit *i* and unit *j* is calculated as
  $$\begin{array}{c} DT\left(i,j\right)=\frac{t_{j}-t_{i}}{\frac{1}{n}\sum_{l=1}^{n}{\left(t_{l}-t_{i}\right)}^{2}} \end{array}$$ (2)
- **Hierarchical position of the alter and difference in hierarchical position between the ego and the alter**: First, for each unit, prestige *h* (which is a vector of length *n*) is calculated as an indicator of a hierarchical level. Prestige is defined as the proportion of the other units that can reach selected ego *i* in two steps by following the directed links. The relative hierarchical position is then calculated for the *i*-th unit as
  $$\begin{array}{c} RH\left(i\right)=\frac{h_{i}}{\frac{1}{n}\sum_{l=1}^{n}h_{l}} \end{array}$$ (3)
  while the difference in hierarchical position between unit *i* and unit *j* is calculated as
  $$\begin{array}{c} DH\left(i,j\right)=\frac{h_{j}-h_{i}}{\frac{1}{n}\sum_{l=1}^{n}{\left(h_{l}-h_{i}\right)}^{2}} \end{array}$$ (4)
- **Popularity level of the alter**: The alter popularity mechanism (below referred to as the “popularity mechanism”) reflects the tendency of creating links to the most popular ones. The popularity statistic (P) is calculated for the *i*-th unit as the ratio between the in-degree of the *i*-th unit and the number of all links in the network:
  $$\begin{array}{c} P\left(i\right)=\frac{\sum_{j=1}^{n}x_{ij}}{\sum_{l=1}^{n}\sum_{j=1}^{n}x_{ij}} \end{array}$$ (5)
- **Outgoing shared partners by the ego and the alter (OSP)**: The outgoing shared partners mechanism is defined through the number of units *k* which are shared partners of the ordered pair (*i*, *j*) if *i* → *k* and *j* → *k*. To compute the statistics associated with this mechanism on selected pair of units *i* and *j*, one must identify the other units (not *i* and not *j*) which are linked with *i* and *j* (shared partners) in a given way:
  $$\begin{array}{c} OSP\left(i,j\right)=\sum_{k\neq i,j}x_{jk}\;x_{ik} \end{array}$$ (6)
  The function gives the number of partners shared by *i* and *j*. By fixing unit *i*, one can obtain vector *V* with *n* elements where each value stands for the number of common partners between unit *i* and all the other units. The *i*-th value of vector *V* can be normalized as $\frac{V_{i}}{\sum_{l=1}^{n}V_{l}}$. Such normalized statistics are used to operationalize the OSP mechanism.
- **Distance between the ego and the alter**: The distance between the ego and the alter is defined by function *G*(*i*, *j*), which returns the minimum number of links needed to reach unit *j* from unit *i* following the directed links. If unit *j* cannot be reached, the function then returns the maximum distance between unit *i* to all other (connected to *i*) units in a network increased by 1. The distance is normalized
  $$\begin{array}{c} DD\left(i,j\right)=\frac{G(i,j)}{\frac{1}{n}\sum_{l=1}^{n}G\left(i,l\right)} \end{array}$$ (7)

Apart from the operationalized local network mechanisms (statistics), the corresponding weights must be chosen. To limit the space of all possible weights’ values, the restriction which is applied is that the sum of the squared values of *θ* equals 1. This implies that the weights can also be negative.

Because the aim of this study is to identify whether the selected local network mechanisms can drive the network towards the chosen global network structure, several different *θ*s are considered. Specifically, 2,000 randomly generated *θ*s are used (see S2 Appendix for more details on generating *θ*s). The *θ*s are generated such that the signs of the weights corresponding to the value-related mechanisms are positive while the signs of the weights corresponding to the cost-related mechanisms are negative. The exception are the weights of the mechanism outgoing shared partners by the ego and the alter, where positive and negative signs are possible. The signs are determined based on the theory previously presented.

### Evaluation of the global network structure

In this study, not only is the emergence of the chosen blockmodel type required, but the hierarchical levels must also be in line with the tenure. Therefore, a two-step evaluation procedure is used.

In step one, the number of inconsistent blocks (e.g., a null block is assumed and a complete block appears) is calculated to evaluate the fit of an obtained global network structure to the ideal chosen one. The definition of the number of inconsistent blocks is based on the blockmodel of the obtained blockmodel (i.e., by using the algorithm) and the blockmodel of the ideal blockmodel (i.e., the assumed blockmodel). Both blockmodels must contain the same number of clusters (in this study, the number of clusters is set to 3). The number of inconsistent blocks is then defined as the number of different types of blocks in both blockmodels. To obtain the blockmodel solution, the same approach is used as in the empirical case (see the Methodology subsection).

In step two, only the results of thetas that produced networks without inconsistent blocks are considered. For the networks produced by these thetas, the average tenure is calculated for each cluster. Only those solutions with zero inconsistent blocks and where the average tenure is increasing along with the hierarchical level are retained.

The amount of inconsistencies can be evaluated by using the relative fit measure (RF) [36, 37]. It is defined as

$$\begin{array}{c} RF=1-\frac{P^{m}}{\frac{1}{k}\sum_{i=1}^{k}P_{i}^{r}} \end{array}$$ (8)

where *k* is the number of randomized networks, *P*^*m*^ is a value of a criterion function of the network of interest (e.g., empirical) and $P_{i}^{r}$ is a value of a criterion function of the *i*-th random network. The mean value of a criterion function for random networks (which is $\frac{1}{k}\sum_{i=1}^{k}P_{i}^{r}$) is estimated by simulations since it depends on many factors (e.g., the algorithm used for generalized blockmodeling, the density …) and therefore cannot be analytically calculated. A higher RF value means a better fit to the ideal blockmodel.

### Results

The nine generated *θ*s (out of 2,000 randomly generated *θ*s) produced networks with the chosen global network structure (all 30 generated networks have the chosen blockmodel type and the average tenure is decreasing with the hierarchical level of the cluster) (see Table 4).

**Table 4:** The selected *θ*s that generate networks without any inconsistent blocks in the blockmodels with the corresponding mean RF.

ID of *θ*	the *θ*s that generated 30 (out of 30) networks without any inconsistent blocks							mean RF	sd of RF
	Hierarchical position of the alter	Tenure of the alter	Popularity level of the alter	Outgoing shared partners by the ego and the alter	Difference in hierarchical position between the ego and the alter	Difference in tenure between the ego and the alter	Distance between the ego and the alter		
1861	0.281	0.380	0.123	-0.016	-0.737	-0.005	-0.468	0.52	0.031
1814	0.041	0.732	0.082	-0.011	-0.612	-0.060	-0.280	0.46	0.042
1757	0.412	0.301	0.197	-0.038	-0.836	-0.008	-0.035	0.45	0.039
147	0.428	0.377	0.002	-0.121	-0.756	-0.026	-0.297	0.44	0.038
1301	0.039	0.590	0.004	-0.096	-0.483	-0.103	-0.630	0.44	0.041
1656	0.339	0.609	0.001	-0.157	-0.600	-0.117	-0.340	0.44	0.048
483	0.001	0.794	0.049	0.085	-0.413	-0.153	-0.406	0.38	0.040
1910	0.376	0.182	0.047	0.032	-0.827	-0.002	-0.371	0.38	0.032
936	0.002	0.651	0.322	0.059	-0.533	-0.246	-0.352	0.37	0.035

The mean RF is between 0.37 and 0.52. It is estimated by the simulation study (see S3 Appendix) that the maximal possible RF value (for the final networks) is approximately 0.59 if the out-degree of each unit is 5 (the mean out-degree in the generated networks is 4.84). This indicates that the highest obtained RF values are very high. The standard deviations of all RF values are low. This indicates that the assumed local mechanisms, especially with the *θ*s with the highest RF, produce networks with the proposed blockmodel type.

RF values are more easily interpreted when calculated for different blockmodel types. Moreover, calculating RF values for different blockmodel types at different steps of their evolutionary process can help to obtain deeper insights into the evolution of the generated networks’ structures. In this study, mean RF values are calculated after each wave of newcomers and at the end of the iterations. The following blockmodels are assumed: cohesive with 3 clusters (the units within each cluster are linked to each other while the units from different clusters are not linked to each other, see Fig 1A), asymmetric core-periphery with 2 clusters (the so-called core units are linked to each other while the “peripheral units” are not linked to each other but are linked to the core ones, see Fig 1G), hierarchical-cohesive with 3 clusters (the units within clusters are linked to each other, units from the third cluster are linked to units from the second cluster and units from the second cluster are linked to units from the first cluster, see Fig 1D) and transitive-cohesive with 3 clusters (similar to the hierarchical-cohesive blockmodel with 3 clusters but here units from the third cluster are also linked to units from the first cluster, Fig 1B).

The cohesive and asymmetric core-periphery blockmodel types are selected because they were found to be present at earlier time points in the empirical networks while the transitive-cohesive blockmodel on the diagonal is selected since it differs in only one or two blocks from the chosen hierarchical blockmodels.

**Fig 8:**
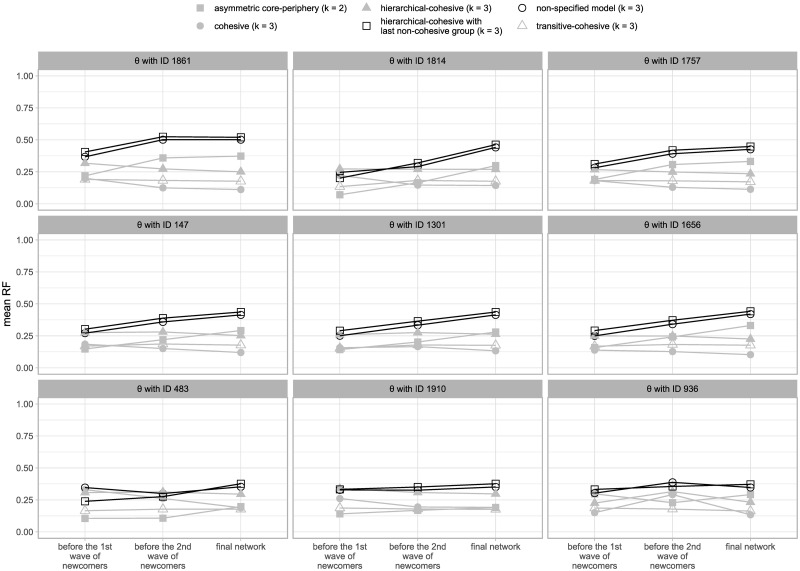
The RF values for the generated networks obtained with different blockmodel types assumed.

**Fig 9:**
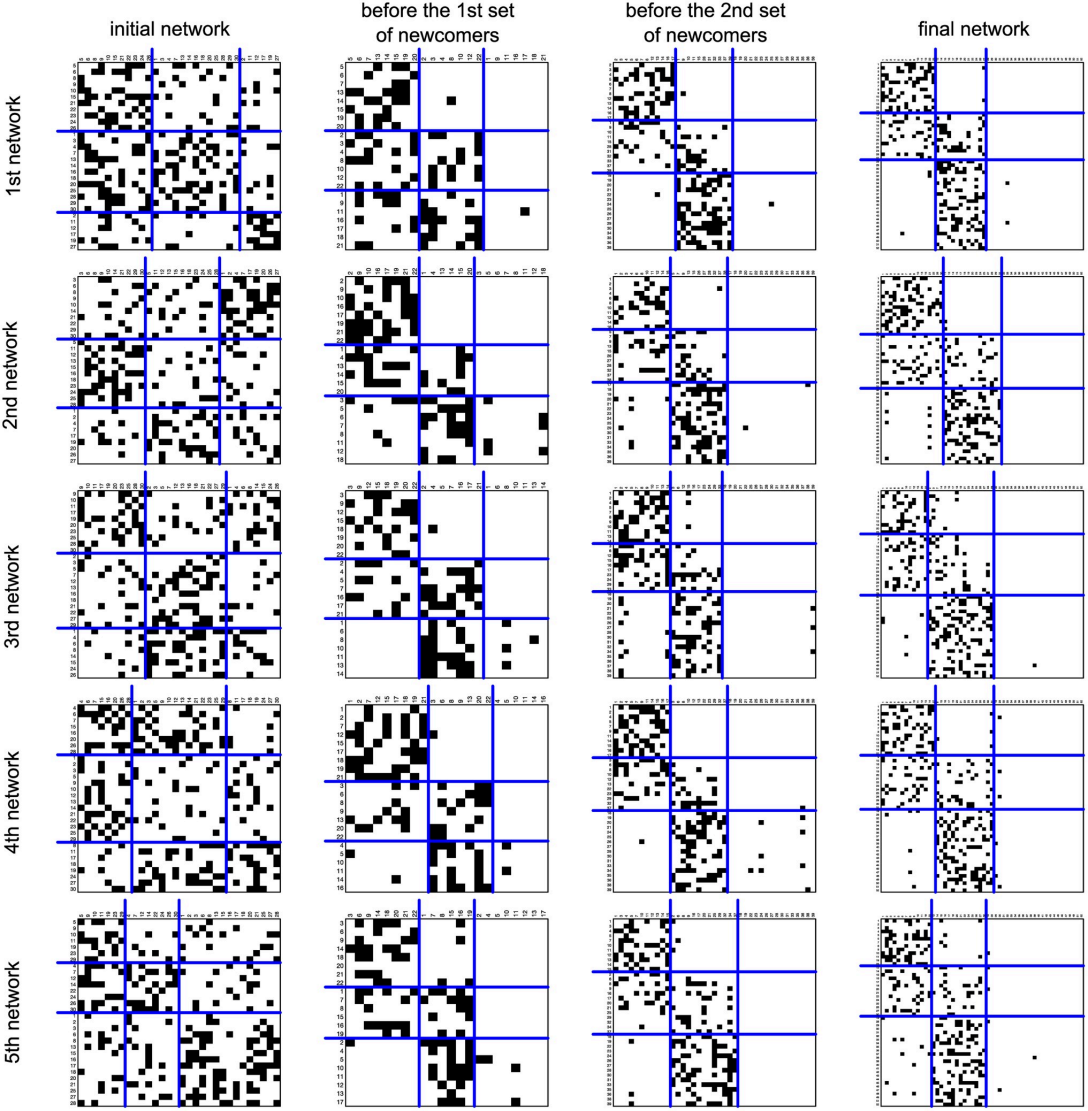
Some generated networks by considering the *θ* with ID 1861. The first five generated networks are shown in lines at different iterations; the networks are drawn in line with the blockmodeling solution for sparse networks, a non-specified model is assumed.

Fig 8 shows the mean RF values calculated on networks generated by the *θ*s shown in Table 4 for different blockmodel types in different phases of the evolutionary process. These phases are determined based on the waves of newcomers where it may be seen that the chosen blockmodel type already begins to form before the first newcomers are added to the network (the mean RF value is highest for the chosen blockmodel at the time before the 1^st^ wave of newcomers arrive), but the structure is less clear as indicated by the mean RF values for other blockmodel types not being considerably lower (a higher amount of inconsistencies can also be seen in Fig 9 with some examples of the networks so generated). The fact the chosen global network structure emerges before the first wave of newcomers shows that tenure might not be necessary for its emergence (because all units have the same tenure prior to the new ones being added to the network).

The next observation in Fig 8 is that the mean RF values for the hierarchical-cohesive blockmodel are close to the mean RF values of the hierarchical-cohesive blockmodel type with the last non-cohesive group (as also seen on the 3^rd^ generated network in Fig 9, there are the cases with links in the last diagonal). Later, the RF values corresponding to the chosen blockmodel and the free blockmodel increase. The values corresponding to the free model are generally slightly lower (than the values corresponding to the chosen blockmodel), which is expected.

Although the mechanisms weights are incomparable, some general conclusions can be drawn with respect to the most extreme values. It may be seen (see Table 4) that the weights for the mechanisms popularity level of the alter, outgoing shared partners by the ego and the alter and difference in tenure between the ego and the alter are generally low (the networks are also generated and evaluated by considering the *θ* with ID 1861, but by setting the weights of the mechanisms popularity level of the alter, outgoing shared partners by the ego and the alter, and difference in tenure between the ego and the alter to 0; the generated networks have a very clear global network structure with the selected blockmodel type).

The weights corresponding to the mechanism tenure of the alter are generally high while the weights corresponding to the mechanism hierarchical position of the alter are in some cases higher and in others lower. When the weights of the mechanism hierarchical position are higher, the weights of the mechanism difference in hierarchical position between the ego and the alter are also generally higher as an absolute value whereas the weight of the mechanism distance between the ego and the alter and the mechanism tenure are generally lower in absolute values.

While positive weights of the mechanism hierarchical position promote links from units on the lower levels to the units on higher levels, the mechanism difference in hierarchical position between the ego and the alter prevent links from those with a very large difference in hierarchical position (e.g., from those on the lowest hierarchical position to those on the highest). Therefore, in order to prevent the emergence of e.g., the transitivity blockmodel, both of these mechanisms must be considered.

The association between the weights of the mechanism hierarchical position and the mechanism distance between the ego and the alter indicates that only one or the other is sufficient for the chosen blockmodel type to emerge (considering all the other local network mechanisms included).

## Discussion and conclusion

By knowing the relationship between the local network mechanisms and global network structures that are entailed, a company can adopt policies to encourage different kinds of communication patterns among the employees to promote the emergence of the global network structure that best suits the flow of knowledge. The decision on which global network structure is most appropriate depends on the type of knowledge to be transferred (e.g., tacit vs. complex knowledge), the type of organization, and its size.

A general research question addresses the transition from the micro to the macro level, i.e. the emergence of a blockmodel under the action-formation type mechanisms and transformational types of local network mechanisms [4]. This general research question is then narrowed to one blockmodel type and the set of local network mechanisms that are commonly discussed within the knowledge-flow context.

The hierarchical-cohesive blockmodel type with the last non-cohesive group was (based on prior studies on this topic) proposed as efficient for knowledge transfer. The existence of this blockmodel type in a real company was shown by analysing the data on knowledge flow in a middle-sized knowledge-based company.

The local network mechanisms of possible relevance for the flow of knowledge among the employees are chosen according to the theory proposed by Nebus [7]. The local network mechanisms are related to the popularity level of the units, their hierarchical level, and the (geodesic) distance between them, the number of outgoing shared partners, and tenure.

The research question was addressed by using the proposed algorithm from the family of network evolution models which considers these mechanisms. The Monte Carlo simulation results show that the chosen blockmodel can be an outcome of the selected local network mechanisms. Probably the most essential mechanisms are those relating to the employees’ tenure and hierarchical position as well as the distance between the employees. These mechanisms operationalize many important social constructs that include different kinds of cost (social, psychological, institutional) and distance (psychic, cognitive, geographical).

The results are especially relevant as they show that the studied global network structure may appear by virtue of some local network mechanisms unrelated to the nodes’ attributes (except for tenure, which can be estimated from the network). This may have several implications. For example, researchers who wish to evaluate approaches for analysing (temporal) networks often strive to generate (temporal) networks not entirely at random, but by some local network mechanisms.

The results are also relevant because they indicate that it is possible to define some general company’s policies to promote the emergence of the desired global network structure of knowledge flow. Nevertheless, when generalizing the results of this study, one has to be aware of several simplifications that were considered. A user must reflect on whether these simplifications are appropriate in a studied social context. The most important simplifications are discussed in the following paragraphs.

- Network complexity: Although the main focus in this paper is on knowledge-flow networks, operationalized by learning and advice networks, one has to be aware that real social networks are multiplex networks, i.e. consisting of different types of relations (formal and non-formal). This implies that the considered mechanisms in this study can be used as a general guideline while designing policies within the company. The complexity of the existing network might need to be considered when designing such policies in a certain social context.
- Knowing the whole network: The next simplification relates to the assumption that the nodes know the whole network structure (know who is asking whom for advice) and the tenure of other employees. This is a very common assumption in many approaches for analysing networks, such as SAOM or ERGM. In the case of the latter models, this assumption is mitigated by considering relevant nodes’ attributes. Because the nodes’ attributes are not considered in this study, one has to estimate in which cases this assumption is not too strict. It is argued here that the assumption is reasonable in the case of smaller networks with a high level of dynamic (communication).
- Operationalization: It is not always easy to operationalize the studied concepts. Many times, a researcher must use a kind of proxy. In the current paper, an example of such operationalization is the operationalization of the concepts physic distance, cognitive distance, geographical distance with geodesic distance. In the simulation study, it would be difficult to find a better operationalization, but in an empirical study where the units’ attributes are available, a better solution could be found.

The results of the Monte Carlo simulation study confirm that the selected local network mechanisms (by considering appropriate weights) can generate the hierarchical-cohesive blockmodel type with the last non-cohesive group which is relevant within the knowledge-flow social context.

## Supporting information

S1 Appendix The algorithm for generating networks. (PDF)

S2 Appendix Generating vectors of the mechanisms’ strengths (*θ*s). (PDF)

S3 Appendix The RF value in an ideal network. (PDF)

S1 Data Network data. A network for the first time point (NET_2004.csv), a network for the second time point (NET_2006.csv), a network for the third time point (NET_2007.csv). Business unit for each unit for the first time point (BU_2004.csv), for the second time point (BU_2006.csv) and for the third time point (BU_2007.csv). Tenure for each unit for the first time point (TE_2004.csv), for the second time point (TE_2006.csv) and for the third time point (TE_2007.csv).
(ZIP)
