# Supplementary material for: Global structures and local network mechanisms of knowledge-flow networks
Source: PLoS One. 2021 Feb 16;16(2):e0246660. doi: 10.1371/journal.pone.0246660 (PMC7886156; doi:10.1371/journal.pone.0246660)
Supplement: S1 Appendix — (PDF) [file pone.0246660.s001.pdf]

The algorithm for generating networks

The network is shown in the form of an adjacency matrix X with n rows and n columns, with both corresponding to the number of units n in the network. There are weights on the links, which are considered only to control the links' duration (see subsection 1.2 in this appendix). The local network mechanisms (see section 1.1 in this appendix and subsection 3.1.2 in the paper) and the global network structures are analyzed by considering binarized networks.

The algorithm allows different initial networks to be specified: either an empty network, random network or a network with a specific global network structure (e.g., a blockmodel). Besides initial network X, parameters λ and κ must be set. Parameter λ express the maximum expected out-degree, while parameter κ is related to the number of iterations (see section 1.2 in this appendix). The local network mechanisms must also be provided with the corresponding vector θ which operationalizes the importance (strength) of the selected local network mechanisms.

The algorithm is iterational. At each iteration, one unit (ego i) is selected among all units in the network (each unit is selected with equal probability)[1]. Considering i and the selected local network mechanisms, the network statistics are calculated and weighted by θ. The weighted network statistics are normalized on the interval between zero and one. Among 25% of the units with the highest weighted network statistics value, one unit is randomly selected.

Further, the tenure is calculated at each iteration and the new units (newcomers, see section 1.3 in this appendix) are added to the network and some existing units are removed (outgoers, see section 1.4 in this appendix) at the selected iterations.

1.1 Weighted network statistics

The weighted network statistics are calculated by the function $compute.S(X,\ i,\ M,\ \theta)$ that considers the set of mechanisms M and the weights of the corresponding mechanisms θ. M is a set of operationalized mechanisms defined on the binarized network X and unit i.

The computed value of a given mechanism (from the set of mechanisms M) is a vector of length n. Each element of such vector corresponds to one unit in the network. When several mechanisms are considered, the vectors can be organized into matrix H with n rows and m columns representing the mechanisms. The matrix so obtained is weighted as $S = H\theta^T$ resulting in a vector of length n which is returned by the function $compute.S(X,\ i,\ M,\ \theta)$.

[1] The probabilities could be different among the units. For example, it could be assumed that those units with a lower tenure will have more opportunities to ask for advice. However, whether this would be a reasonable assumption it depends on the company's policies and organizational culture. To consider the most parsimonious case, it is assumed, in this study, that all the units have equal probabilities to ask for advice at any time.

1.2 Duration of the links

No specific mechanism considered in this study would control the duration of a link (i.e. duration of the advice seeker–advice giver interaction). Instead, it is assumed that all interactions last the same amount of time. One unit of time is defined through the number of iterations at which each unit will receive (on average) the selected number (λ) of opportunities to establish a link. The number of iterations depends on the network's size and the desired maximum expected out-degree (parameter λ).

Let us consider a case without newcomers and outgoers. Further, let us assume there are n units in the network and each unit has up to λ opportunities to establish (or confirm) a link (the loops are not considered). On the assumption that the units are chosen randomly with equal probability, the number of iterations needed to reach the expected number of opportunities to establish a link is $\alpha = \lambda n$ (namely, the length of one unit of time). In α iterations, each unit gets (on average) λ opportunities to create a link. This implies that some individual units can have a higher out-degree, which happens because some receive more opportunities to establish a link than others and because some units can establish more links to the same others while some other units can establish links to a higher number of other units. In a very unlikely case, each unit could receive exactly λ opportunities to create a link. In that case, the maximum out-degree of each unit would be $min(n-1,\ \lambda)$ if no tie is chosen twice (in a sense confirmed).

The links last a limited amount of time. Specifically, the link duration is set to $\alpha + 1$ iterations. When new units are added to the network, parameter α must be updated by considering the new number of units. This implies that the number of iterations between different waves can wary. The algorithm is implemented in such a way that the number of outgoers does not affect the number of iterations.

In order to ensure enough iterations so that the considered mechanisms can affect a global network structure considerably, the number of iterations is multiplied by the constant κ. The value of $\kappa > 1$ increases the expected number of opportunities for each unit to establish a link while it does not affect the duration of a link and the maximal expected out-degree. A higher expected number of opportunities for each unit to create a tie can also increases the structure's stabilization before the new units are added. In other words, a higher number of iterations gives "more time" to the mechanisms to affect the global network structure before the newcomers are added to the network.

1.3 Newcomers

New units can be added one by one or in several waves. The iterations upon which the new units are added to the network can be selected in different ways: (i) one unit can be added at a time; or (ii) a group of units can be added all at once. Further, the unit (or groups of units) can be added at randomly selected iterations or be added at predefined iterations e.g., equally distributed across the iterations. In this study, newcomers are added in three waves. The number of newcomers for each wave is represented by vector $\aleph$. The number of iterations between each wave is determined based on the total number of units in the network, based on parameter λ and parameter κ.

1.4 Outgoers

The number of outgoers can be selected arbitrarily. They can leave the network in waves just before or after newcomers are added or can leave the network one by one. With this implementation of the algorithm, the outgoers leave the network at the selected iterations in vector O. The units to be removed from the network can be selected based on their personal characteristics (e.g., tenure), network characteristics (e.g., popularity or hierarchical level), or randomly. Here, the units to be removed are randomly selected, which is in line with the observations on the empirical data. The number of units to be removed from the network is 25% of all units in the network calculated immediately after a wave of newcomers has been added to the network.

Algorithm 1 The algorithm for generating networks

```
import initial network X (a matrix with n rows and n columns, where n is the number of units)
import θ (a vector with the mechanisms' weights)
import M (a set of functions which defines the mechanisms)
set λ (the expected maximum out-degree)
set κ (the factor by which the number of iterations must be increased between the waves)
set ℵ (a vector with the number of newcomers per waves)
set O (a vector with iterations at which the outgoings are to be removed)
set T (tenure, a vector of length n)
compute forTenureCorVec = cumsum(ℵ_A) * λ * κ (the number of iterations between consecutive waves
of newcomers)
compute E = cumsum(forTenureCorVec) (iterations at which the newcomers are added to the network)
compute k = max(E) (the total number of iterations)
set forTenureCor to first element of forTenureCorVec
for l in 1:k
|__| set T = T + 1/forTenureCor
|__| randomly select a unit i (actor/ego)
|__| calculate S = compute.S(X_l, i, M, θ) (a vector of the weighted network statistics with the
length n)
|__| calculate φ = (S − min(S)) / (max(S) − min(S)) (normalize S, so the min(S) = 0 and max(S) = 1)
|__| if φ ≥ Q_3(φ) then classify a corresponding unit into set C (where Q_3 is the third quartile)
|__| randomly select unit j among the units from set C
|__| set a link i → j
|__| calculate X = X − 1/(λn + 1)
|__| calculate X = {0, x ≤ 0
                   {x, x > 0
| if l ∈ O
|_________|randomly select a unit or a group of units to be removed
|_________|remove the selected unit(s) and update X and T accordingly
|if l ∈ E and l ≠ k:
|_________|add a unit or a group of units and update X and T accordingly
|_________|set forTenureCor to next element of forTenureCorVec
return network X
```
