# Supplementary material for: Global structures and local network mechanisms of knowledge-flow networks
Source: PLoS One. 2021 Feb 16;16(2):e0246660. doi: 10.1371/journal.pone.0246660 (PMC7886156; doi:10.1371/journal.pone.0246660)
Supplement: S2 Appendix — (PDF) [file pone.0246660.s002.pdf]

Generating vectors of the mechanisms' strengths (θs)

θ is a vector of length corresponding to the number of local network mechanisms being considered. To limit the space of all possible values, the restriction $\sum \theta_i{}^2 = 1$ is applied. Individual θs may be seen as points in the space. When each θ consists of three values, the points are distributed across the sphere. There are several approaches to generating random points on the sphere and can be generalized on a higher dimension. One approach is to generate the values from the standard normal distribution Φ and multiply them by a scalar, $\theta = \Phi\left(1/\sqrt{\sum \Phi_i}\right)$ (Marsaglia, 1972; Muller, 1959). The values (points) are randomly distributed when this approach is applied, but some are much closer to each other than others. To solve this issue, several approaches have been proposed, but all are limited to the three-dimensional space.

Figure 1 Example of some generated θs by the two different approaches

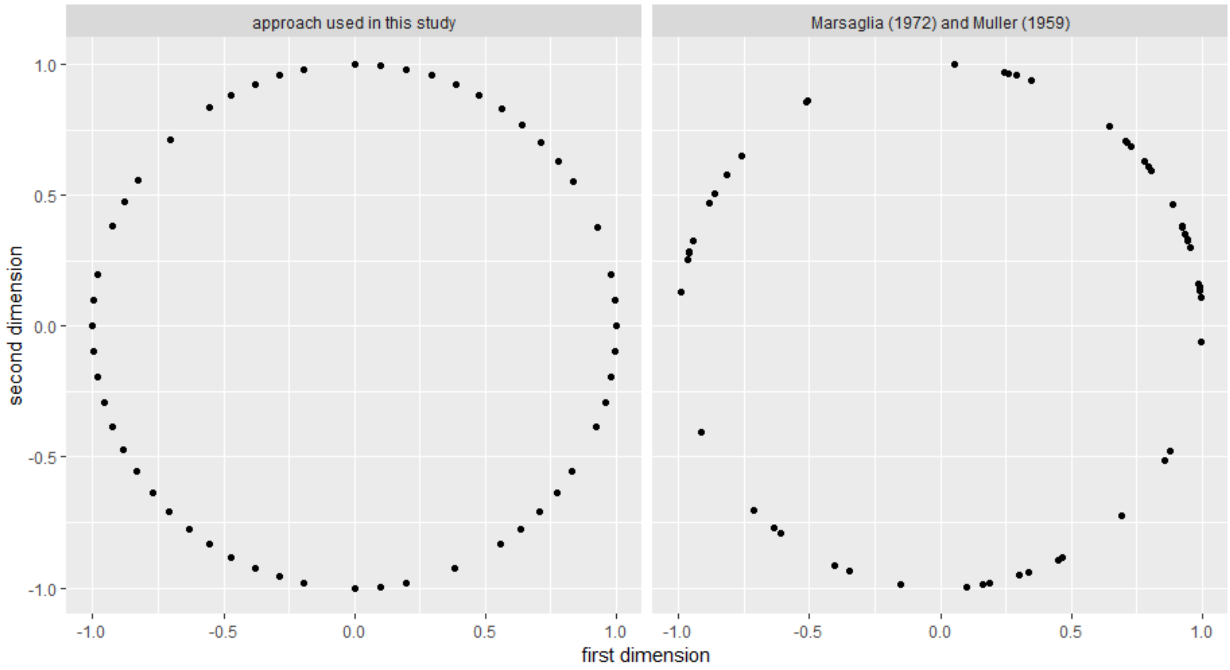

Therefore, the following approach for generating random points in n-dimnesional space is used in this paper. When this approach is applied, the points are relatively equally distributed. The algorithm is as follows: The user must set the desired number of θs (points) to be generated as well as the initial set of θs, I. The iterative process is then initiated. In this process, the approach taken by Marsaglia (1972) and Muller (1959) is used to generate a set of θs called C, which are the candidates for being added to the initial set I. The number of elements of set C is arbitrary, but the highest number results in more equally distributed points. Then, the θ from set C with the maximal minimal distance to any θ from set I is identified and added to set S.

As illustrated in Figure 1, the points are more equally distributed when using the described approach than the approach proposed by Marsaglia (1972) and Muller (1959) (54 θs are generated by each approach, each set C consists of 1,000 θs).
