# Supplementary material for: Global structures and local network mechanisms of knowledge-flow networks
Source: PLoS One. 2021 Feb 16;16(2):e0246660. doi: 10.1371/journal.pone.0246660 (PMC7886156; doi:10.1371/journal.pone.0246660)
Supplement: S3 Appendix — (PDF) [file pone.0246660.s003.pdf]

The RF value in an ideal network

The amount of errors (inconsistencies) between different networks with the same blockmodel is evaluated by using the relative fit measure (RF), defined as

$$RF = 1 - \frac{P^m}{\frac{1}{k}\sum_{i=1}^{k} P_i^r}$$

where k is the number of randomized networks, P^m is the value of a criterion function of the network of interest (e.g., empirical) and P_i^r is the value of a criterion function of the i-th random network. The criterion function for structural equivalence (Doreian, Batagelj, & Ferligoj, 2005) is defined for nondiagonal blocks as

$$\delta(R,B) = \sum_{x \in C_u,\ y \in C_v} \left| r_{xy} - b_{xy} \right|$$

where R corresponds to the observed nondiagonal block and B to the ideal block. Next, r_{xy} is the observed link and b_{xy} is the corresponding value in the ideal block. In line with the generalized blockmodeling approach for sparse networks (Žiberna, 2013), the errors in null and complete blocks can be weighted differently. In this study, complete blocks are weighted by $d/(1-d)$ and null blocks are weighted by 1, where d is the density of the whole network. When there are no inconsistencies (i.e., no links in null blocks and all links in complete blocks), the value of one or the other criterion function would equal 1. But the number of outgoing links is constrained by the algorithm for generating networks (which is an operationalization of the restricted ability to maintain an infinite number of relationships) to the number which is lower than that implied by the chosen blockmodel type with three groups. This means it is theoretically impossible to have the value of a criterion function equal to 1.

Figure 1 Distribution of the RF values corresponding to 10,000 randomly generated networks in line with the chosen blockmodel

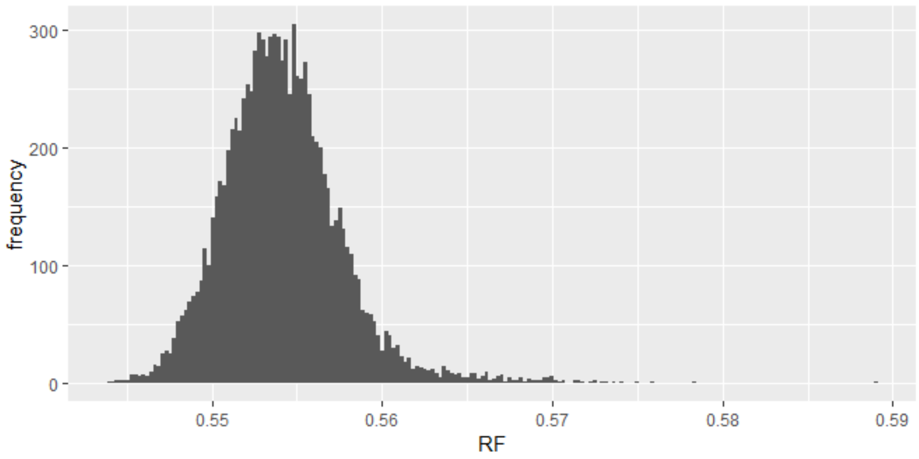

The simulation study was applied to estimate the expected and maximum value of a criterion function used in the blockmodeling approach for sparse networks. For this purpose, 10,000 random networks were generated in line with the chosen blockmodel in such a way that there were no links in null blocks and the out-degree of each unit was 5. This is very close to the best theoretical fit of the global network structure to the chosen blockmodel that could be achieved by considering the constraints of the out-degree. The blockmodeling approach for sparse networks was used on each generated network and the value of the criterion function was calculated.

The RF values were also calculated for all generated networks. The mean value of a criterion function for the case of random networks was estimated by simulations. To this end, 30 random networks (with the same density as in ideal networks) were generated for each of 10,000 generated random networks with the chosen blockmodel.

The distribution of the RF values for all generated networks is given in Figure 1. The minimum RF value is 0.53 and the maximum is 0.59, while the mean value is 0.55. RF values close to 0.59 obtained on the generated networks are considered to be high.
